# Measuring economic burden in families of individuals with Angelman Syndrome in Poland: a caregivers’ survey

**DOI:** 10.1186/s13023-025-04183-4

**Published:** 2025-12-30

**Authors:** Dariusz Walkowiak, Karolina Pospieszyńska-Martysiuk, Hanna Dianow, Joanna Węgrzyn, Jan Domaradzki

**Affiliations:** 1 Department of Organization and Management in Health Care, Poznan University of Medical Sciences, Poznan, Poland; 2 FAST POLAND – Fundacja Foundation for Angelman Syndrome Therapeutics Poland, Gdansk, Poland; 3 Department of Social Sciences and Humanities, Poznan University of Medical Sciences, Rokietnicka 7, Poznan, 60-806 Poland

**Keywords:** Angelman syndrome, Cost-of-illness, Financial burden, Economic evaluation, Health expenditures, Healthcare utilization, Income loss, Out-of-pocket expenses, Rare diseases

## Abstract

**Background:**

Angelman Syndrome (AS) is a rare neurogenetic disorder characterized by severe intellectual disability, seizures, and motor and speech impairments, requiring lifelong, intensive care. Although caregivers of individuals with AS often face reduced quality of life and substantial emotional and financial strain, little is known about the cost-of-illness in Poland. To address this gap, we conducted a cross-sectional study in collaboration with the Foundation for Angelman Syndrome Therapeutics Poland (FAST Poland). An anonymous web-based survey was completed by 85 parents of individuals with AS to assess the 12-month financial burden of caregiving, including healthcare utilization, non-medical expenses, caregiver-related health costs, and broader socioeconomic impacts.

**Results:**

The average annual out-of-pocket cost per household was $69,511, which far exceeded the average public reimbursement of $46,928. Major private expenditures included therapy and rehabilitation ($7,836), non-reimbursed rehabilitation camps ($4,451), and medical equipment ($2,637). Income loss due to caregiving averaged $31,809 per household, contributing significantly to the overall burden. Psychological support and medication for caregivers generated additional costs, often paid entirely out of pocket. Spearman’s correlations revealed that younger patients incurred higher hospitalization and private rehabilitation expenses, while older individuals required more home modifications. Caregiving averaged 89.4 h per week, with minimal professional or informal assistance. Despite most families receiving public support, it did not offset the full economic impact, particularly indirect costs such as reduced employment. The findings underscore the chronic, high-cost nature of AS caregiving in Poland, marked by insufficient systemic support and reliance on family-provided, unpaid care.

**Conclusion:**

AS imposes a major financial burden on families, as out-of-pocket expenses and income loss surpass public aid. These findings underscore the urgent need for improved systemic and policy-level support for caregiving.

## Background

Angelman Syndrome (AS) (ICD-10: Q93.51, ICD-11: LD90.0, ORPHA: 72, OMIM: 105830) is a rare neurogenetic disorder [1, 2]. With a prevalence of approximately 1 in 12,000 to 20,000 individuals, it is estimated that AS affects approximately 500,000 people worldwide [3, 4], including 145 individuals registered in the Global Angelman Syndrome Registry from Poland [5]. AS results from several distinct genetic mechanisms, most commonly a maternal 15q11.2–q13 deletion (70–75%), followed by pathogenic variants in the maternal *UBE3A* gene (~ 15%), paternal uniparental disomy (5–7%), and imprinting defects (5–7%); mosaicism occurs rarely and is usually associated with an epimutation [1]. These genetic differences significantly influence both the clinical severity and caregiving demands related to the condition [6].

While different genetic mechanisms may lead to distinct phenotypic variations, all forms of AS share core clinical features [7, 8]. The syndrome is typically marked by severe intellectual disability, motor and balance impairments, absent or minimal speech, epilepsy, and sleep disorders [9–12], along with craniofacial features like microcephaly, prognathism, macroglossia, and deep-set eyes [9, 13, 14]. A distinct behavioral phenotype includes frequent laughter, hand flapping, tremors, hyperactivity, attention difficulties, feeding problems, drooling, and aggressive or self-injurious behaviors, as well as a fascination with water [9, 13, 15]. Common comorbidities include scoliosis, gastrointestinal symptoms, and autism spectrum disorder [11, 16].

Due to its heterogeneous presentation, AS is often misdiagnosed, prolonging the diagnostic process [10, 16–20]. Therefore, confirmation of the diagnosis requires genetic testing (e.g., methylation analysis, MS-MLPA, microarrays, FISH, or *UBE3A* gene sequencing) [14, 21]. Although there is no curative treatment, research is ongoing, and current care is supportive, involving medication, rehabilitation, and multidisciplinary therapies [22–24]. Consequently, in addition to regular rehabilitation and, frequently, hospital stays, people with AS need continuous, comprehensive, multidisciplinary home-based care [10, 25]. Simultaneously, while life expectancy in AS is generally normal, risks such as sudden unexpected death in epilepsy (SUDEP) and sudden unexpected death in sleep (SUDS) may shorten it by 10–15 years [26–28].

For all these reasons, families must provide constant, intensive support, often leading to physical (e.g., fatigue, injury), mental (e.g., depression, anxiety), and social burdens (e.g., isolation) [29–33]. As a result, caregivers often experience stress, lower quality of life, and greater psychological vulnerability than those caring for children with other neurodevelopmental or rare syndromes [34–37], frequently requiring psychiatric care [10, 16–20]. Financial strain is also considerable and includes frequent visits to healthcare professionals, the need for health-related consumables, varying out-of-pocket expenses, and disparities in insurance coverage depending on the national healthcare system. Many parents are compelled to reduce their working hours or leave the workforce entirely [6, 10, 30, 32, 34, 38–40]. Studies consistently show that AS imposes substantial economic burdens on families, driven by both direct costs (e.g., therapies, equipment, home modifications) and indirect costs (notably caregiver productivity loss). For example, in the U.S., the average annual cost per individual with AS was estimated at nearly $80,000, with over half attributed to lost productivity [41]. In Australia, parents of individuals with AS were found to lose over 38% of their productivity-adjusted life years, resulting in a societal cost of AUD$45.3 million, with mothers bearing the majority of the burden due to reduced employment [39]. These financial strains are compounded by the negative impact of caregiving on parents’ physical and mental health, further affecting their quality of life and economic stability.

Despite growing international knowledge, little is known about the specific cost-of-illness associated with AS in Poland. Recent studies suggest that while diminished quality of life and caregiver burden among parents of children with AS are often linked to the diagnostic odyssey and its perceived impact on the child’s health, financial well-being is the strongest single predictor of reduced quality of life and increased burden in caregivers [33]. Therefore, this study aims to evaluate the cost-of-illness related to caregiving for individuals with AS in the Polish context.

## Methods

### Study design

Given the sensitive and socially significant nature of the study, it was designed as a patient-oriented survey [42, 43] and was developed in collaboration with the patient advocacy organization Fundacja Foundation for Angelman Syndrome Therapeutics Poland – FAST Poland (https://www.cureangelman.pl), which actively participated in all phases of the study’s design and implementation. This ensured that the study addressed caregiver-identified priorities and enabled active engagement of caregivers as research partners.

In cooperation with the Foundation, an anonymous, computer-assisted, web-based self-reported survey was developed using Microsoft Forms (Microsoft 365 platform) to assess the direct and indirect financial costs of caring for a person with AS, including healthcare utilization, non-medical expenditures, health-related costs for caregivers, and the broader social and economic burdens on families.

The study was approved by the Bioethics Committee of the Poznan University of Medical Sciences (KB – 660/24, issued on October 9, 2024) and received additional acceptance from the board of FAST Poland.

### Participants

Because AS is a rare condition and Poland lacks a national patient registry, FAST Poland assisted in participant recruitment by inviting caregivers through internal communication channels. Given the absence of national epidemiological data, as no authoritative national rare disease registry exists in Poland, and there are no precise epidemiological data on the prevalence of AS in the Polish population, the target sample size could only be estimated pragmatically. Eligible participants were included if they were over 18 years old, were parents or primary family caregivers of an individual with a molecularly confirmed diagnosis of AS, were Polish, had access to the Internet Patient Account (Internetowe Konto Pacjenta – IKP) system, were able to use electronic devices for online participation, and provided written informed consent.

### Questionnaire

The development of the questionnaire was guided by a literature review and a focus group interview with three mothers of children with AS. In addition, the structure of cost categories was informed by established cost-of-illness frameworks and previous economic evaluations in AS and other rare neurogenetic disorders [39–41, 44–46]. The tool aimed to evaluate caregivers’ assessment of the cost-of-illness in AS and the health expenditures associated with caregiving.

A pilot test was conducted with five parents to evaluate the questionnaire’s clarity and relevance, and these pilot responses were not included in the final analysis. The survey retrospectively explored how caregiving affected respondents in several key areas: (1) direct medical expenses related to caring for a person with AS in the past 12 months (January 1 to December 31, 2024), whether covered by the National Health Fund (Narodowy Fundusz Zdrowia – NFZ) or paid out-of-pocket (e.g., hospitalizations, medications, specialists’ visits, rehabilitation and therapy, medical equipment, etc.); (2) indirect medical expenses (e.g., travel, private services, costs of parent’s stay during hospitalization, etc.); (3) health-related costs for caregivers over the same 12-month period, including both reimbursed and privately funded psychological and psychiatric care, as well as any additional health-related expenditures; and (4) direct and indirect non-medical expenses (lost income due to job resignation, extra electricity, heating, water, home modifications, additional insurance). Additionally, the questionnaire included questions on caregiver demographics and the demographic and clinical profile of the person with AS, including age, sex, clinical symptoms, comorbidities, and general medical condition.

Clinical characteristics, symptoms, and comorbidities of individuals with AS were collected through caregiver self-report as part of the structured online questionnaire. Respondents provided information based on their own knowledge, supported where possible by data retrieved from the Internet Patient Account (IKP) and their personal medical documentation. The list of symptoms and comorbidities was informed by previous AS studies, international clinical descriptions (ICD, OMIM, Orphanet), and consultation with FAST Poland, but was not derived from a formal AS disease concept model.

The questionnaire was filled out retroactively by participants, who entered information from the IKP system to assess the impact of caring during the previous 12 months.

### Data collection

Data were collected between January and February 2025 via FAST Poland’s internal mailing lists, social media platforms, and official website. In the initial phase, participants received an invitation letter introducing the research team and outlining the study’s aims, methodology, and plans for data dissemination. The letter also highlighted the voluntary, anonymous, and confidential nature of the survey.

Next, all eligible participants were provided with instructions on how to navigate the IKP system and complete the questionnaire. Advance notice of the study’s scope enabled participants to prepare information for the economic evaluation of their caregiving experiences, either by locating relevant receipts, notes, or records documenting privately funded procedures or by retrieving data from their IKP account. Access to the web-based questionnaire was granted after participants submitted electronic informed consent, in accordance with the Declaration of Helsinki. No financial incentives were offered for participation. To avoid duplicate reporting, only one caregiver per household was included, and each questionnaire represented a single household.

### Data analysis

Descriptive statistics were calculated to summarize demographic characteristics, service utilization, and cost data. Continuous variables are presented as means with standard deviations (SD), medians, and ranges, while categorical variables are reported as counts and percentages. Associations between patient age and both reimbursed and out-of-pocket service utilization or expenditures were assessed using Spearman’s rank correlation coefficient (ρ), with corresponding 95% confidence intervals (CI). All analyses were two-tailed, and statistical significance was set at *p* < .05. Analyses were conducted using JASP software (version 0.19.3; JASP Team, Amsterdam, The Netherlands).

All cost data were initially collected in the local currency (Polish złoty, PLN), but are presented in international dollars ($) in the tables. Currency conversion was based on Purchasing Power Parity (PPP) using 2024 rates, with an exchange rate of 1 $ = 1.9 PLN (estimate as of June 17, 2025), in line with the methodology of the International Monetary Fund [47]. PPP is an economic approach that compares the relative value of currencies by measuring the amount of goods and services they can purchase. It adjusts for price level differences between countries, allowing for a more accuratAdditionally, the questionnairee comparison of economic indicators like income and expenditure. By using PPP, we account for local cost-of-living variations, enabling a comparison of economic data based on real purchasing power rather than nominal exchange rates.

## Results

Because national epidemiological data on AS are unavailable, it was not possible to determine the exact proportion of Polish families represented in this sample. A total of 85 caregivers participated in the survey. According to internal estimates from FAST Poland, the age and gender distribution of our survey population appears broadly consistent with that of the known Polish AS community, suggesting that the sample is reasonably representative in these key demographic characteristics. However, caregivers of the three oldest individuals in the Registry (aged 40, 33, and 32 years) did not participate, likely due to limited use of the Internet Patient Account (IKP) system among older caregivers. Most caregivers were female (92.9%), with a mean age of 39.4 years, and 45.9% were professionally active (Table 1). Individuals with AS had a nearly equal sex distribution and a mean age of 8.7 years. The most common genetic diagnosis was a 15q11–q13 deletion (80%). Nearly all patients exhibited core AS symptoms such as psychomotor delay (97.6%), intellectual disability (94.1%), speech impairment (95.3%), motor and balance disorders (97.6%), and abnormal muscle tone (96.5%). Frequent comorbidities included ophthalmologic (72.9%), orthopedic (74.1%), gastrointestinal (68.2%), behavioral, and sensory issues, including, short attention span (92.9%), sensory integration difficulties (88.2%), sleep disturbances (84.7%), hyperactivity (77.6%), excessive biting or oral self-stimulation (77.6%), and features associated with autism spectrum disorder (41.2%). In the year before the survey, 51.8% of parents reported varying frequencies of seizures in their children. Additionally, the diagnostic process was often prolonged and complex: while the median time to obtain a correct diagnosis was 1.5 years, 48.2% of patients received at least one misdiagnosis.

**Table 1:** Demographic characteristics of the survey population

Characteristics	*N* (%)
**AS caregivers**	
*Sex*	
female	79 (92.9)
male	6 (7.1)
*Age (in years)*	
Range	29–55
Mean (SD)	39.4 (6.3)
95%CI for mean	38-40.7
Median	39
*Professional activity*	
yes	39 (45.9)
no	46 (54.1)
**AS patients**	
*Sex*	
female	42 (49.4)
male	43 (50.6)
*Age (in years)*	
Range	1–26
Mean(SD)	8.7 (5.4)
95%CI for mean	7.5–9.9
Median	8
*Genotype determined at diagnosis*	
deletion of the 15q11-q13 region	68 (80)
*UBE3A* gene mutation	8 (9.4)
uniparental disomy (UPD)	4 (4.7)
imprinting centre defect	2 (2.4)
other	3 (3.5)
*Symptoms present in a person with AS*	
delayed psychomotor development	83 (97.6)
intellectual disability	80 (94.1)
severe speech impairment or complete lack of verbal communication	81 (95.3)
seizures	67 (78.8)
motor and/or balance disorders (i.e., ataxia, coordination problems)	83 (97.6)
muscle tone disorders	82 (96.5)
strabismus, other ophthalmologic problems	62 (72.9)
orthopaedic problems (i.e., scoliosis, contractures, foot abnormalities)	63 (74.1)
gastrointestinal disorders (reflux, constipation)	58 (68.2)
feeding/eating difficulties and/or challenges (e.g., food selectivity, ARFID, etc.)	35 (41.2)
excessive biting/oral self-stimulation, including use of inedible objects	66 (77.6)
sucking/swallowing (dysphagia) / chewing difficulties	51 (60)
overweight / obesity	19 (22.4)
abnormal sleep-wake cycles and sleep disturbances	72 (84.7)
hyperactivity, restlessness	66 (77.6)
atypical or inappropriate behaviour (i.e., excessive laughter)	55 (64.7)
short attention span	79 (92.9)
autism spectrum disorders	35 (41.2)
sensory integration disorders	75 (88.2)
self-injurious or aggressive behaviour	30 (35.3)
*Did the patient have seizures in the past year?*	
yes, less than 10	23 (27.1)
yes, between 10 and 20	9 (10.6)
yes, over 20	12 (14.1)
no	41 (48.2)
*Time spent before the correct diagnosis was made(in years)*	
*Range*	0.1–15
*Median*	1.5
*Mean(SD)*	2.5(3)
*Number of misdiagnoses*	
0	41 (48.2)
1	22 (25.9)
2	11 (12.9)
3	4 (4.7)
4	4 (4.7)
5+	3 (3.5)

Table 2 summarizes the specialist medical and therapeutic services used by individuals with AS in 2024. Nearly all patients received care from neurologists (95.3%) and paediatrician or general practitioners (89.4%), with frequent consultations from rehabilitation physicians (69.4%), ophthalmologists (63.5%), and dentists (65.9%). Psychological support was accessed by 55.3% of patients, while visits to psychiatrists, dietitians, and other specialists were less common (23.5%, 18.8% and 11.8%, respectively).

All individuals received physical rehabilitation, with high use of speech therapy (91.8%), AAC methods (71.8%), sensory integration (82.4%), and occupational therapy (60%). Psychological therapy (57.6%), hydrotherapy (50.6%), and animal-assisted therapy (45.9%) were also notable. Less common were manual therapy, osteopathy, and feeding therapy.

**Table 2:** Specialist medical and therapeutic services utilized by individuals with AS in 2024 (January 1, 2024–December 31, 2024)

Characteristics	*N* (%)
*Specialist care received by the person with AS during the past year*	
neurologist	81 (95.3)
geneticist	27 (31.8)
psychologist	47 (55.3)
psychiatrist	20 (23.5)
orthopaedist	34 (40)
dietitian	16 (18.8)
gastroenterologist	17 (20)
ophthalmologist	54 (63.5)
otolaryngologist (ENT specialist)	37 (43.5)
rehabilitation physician	59 (69.4)
gynaecologist	3 (3.5)
dentist	56 (65.9)
cardiologist	10 (11.8)
paediatrician / primary care physician (GP)	76 (89.4)
other	
*Types of therapy utilized by the person with AS during the past year*	
physical rehabilitation/physiotherapy	85 (100)
psychological therapy	49 (57.6)
occupational therapy	51 (60)
speech / neurospeech therapy	78 (91.8)
augmentative and alternative communication (AAC)	61 (71.8)
feeding therapy	9 (10.6)
manual therapy	29 (34.1)
sensory integration therapy	70 (82.4)
sleep training	0
osteopathy	18 (21.2)
animal-assisted therapy (zootherapy)	39 (45.9)
hydrotherapy, swimming/water therapy (with or without hydrotherapy)	43 (50.6)
other	10 (11.8)

Table 3 outlines the socioeconomic status and caregiving support in households of individuals with AS. Most families had only one income earner (63.9%), and 36.5% reported having no cash savings, while 51.8% were regularly repaying debts or other financial obligations. At the same time, 96.3% of families reported receiving financial support from the government and local government institutions, with an average amount of $19,745.

Caregiving demands were high, averaging nearly 90 h per week (median: 97 h), while external support was minimal; both professional and informal care averaged close to zero, at 3.9 and 5.7 h per week, respectively. These findings highlight families’ strong dependence on unpaid, family-based care with little outside assistance.

**Table 3:** AS household socioeconomic characteristics and caregiving support

Characteristics	*N* (%)
*Number of household members with regular income*	
0	1 (1.2)
1	56 (63.9)
2	28 (32.9)
*The household has cash savings*	
yes	54 (63.5)
no	31 (36.5)
*The household has instalments*,* loans*,* debts*,* credits*,* or arrears* (e.g., rent, electricity, telephone, back taxes) to pay?	
yes, but we are repaying them regularly	44 (51.8)
yes, and we’re having trouble repaying them	6 (7)
no	35 (41.2)
*Number of hours per week spent on caregiving*	
Range	2-168
Median	97
Mean(SD)	89.4 (45.2)
*Number of hours per week of professional carer (e.g.*,* nurse*,* social worker*,* disability assistant*,* etc.)*	
Range	0–45
Median	0
Mean(SD)	3.9 (9.3)
*Number of hours per week of unpaid care from informal carers (e.g.*,* family member*,* distant relative*,* neighbours*,* volunteer)*	
Range	0–68
Median	0
Mean(SD)	5.7 (11.4)
*Total monetary value of assistance from the government and local government institutions*	
number of caregivers who received support	82 (96.3)
the average amount of financial support received	US$19,745

Table 4 presents annual healthcare expenditures for individuals with AS and their caregivers in 2024, divided into public reimbursements and out-of-pocket costs. Public healthcare expenditures reimbursed by the NFZ were reported for all 85 patients, with the most common reimbursements covering medications (91.8%, $708 annual cost per patient), hospitalizations (60%, $9,162), and rehabilitation or therapy (63.5%, $3,159). Other reimbursed services included specialist visits (75.3%, $699), professional caregiving (36.5%, $2,263), rehabilitation camps (17.7%, $312), and medical equipment (62.4%, $1,032). Car-related expenses were reimbursed for 21.2% of patients ($437). Caregiver-related reimbursements included psychological or psychiatric care (38.8%, $267), rehabilitation (15 individuals, $14), and psychiatric medications (15 individuals, $19). The total average reimbursed cost per patient was $46,928. Additionally, while nearly all children attended special needs schools, the average educational costs reimbursed by the government and local government institutions totaled $28,856.

Out-of-pocket expenditures placed a heavy burden on AS families. Common costs included supplements (97.6%, $982 annual cost per-patient), therapy and rehabilitation (88.2%, $7,836), and medical equipment (85.9%, $2,637). Over half of patients (55.3%) attended non-reimbursed rehabilitation camps ($4,451 per patient), and 81.2% had private specialist visits ($903). Other frequent expenses (83.5%) included food, legal fees, and miscellaneous items ($2,154). Caregivers incurred additional costs for psychological support (32.9%, $617), rehabilitation (49.4%, $536), and psychiatric medications (15.3%, $212).

Indirect costs added to the economic burden of caregiving: transport expenses affected 88.2% of households ($2,126 annual cost per-patient), and 65.9% experienced job-related income loss averaging $31,809. Informal care was valued at $6,207 annually in 48.2% of households, and 54.1% reported home modification costs ($4,292). Other indirect expenses included increased utility bills (89.4%, $447), caregiver support (58.8%, $1,109), and insurance (40%, $505).

Overall, the average annual out-of-pocket cost per household was $69,511, highlighting the substantial economic impact of AS beyond publicly funded care.

**Table 4:** Annual healthcare expenditures for AS patients and caregivers

Category	Patients *N* (%)	Value per patient (US$)
**Average annual public healthcare expenditure in 2024 (January 1**,** 2024-December 31**,** 2024)**,** reimbursed by the National Health Fund**,** the government**,** and local government institutions**		
***Reimbursed direct medical costs for AS patient***		
hospitalizations	51 (60%)	9,162
medications	78 (91.8%)	708
specialists’ visits	64 (75.3%)	699
rehabilitation and therapy	54 (63.5%)	3,159
paid professional care, e.g., nurse, social worker, disability assistant, etc.)	31 (36.5%)	2,263
rehabilitation camps	15 (17.7%)	312
medical equipment, e.g., wheelchairs, medical beds, walking frames, walkers, standers, orthoses, orthopaedic shoes, etc.	53 (62.4%)	1,032
car customization/purchase	18 (21.2%)	437
increased educational costs related to fulfilling compulsory schooling requirements, associated with a severe degree of disability.	83 (97.6%)	28,856
***Reimbursed direct medical costs for the caregiver***		
psychiatrist or psychologist support for the caregiver	33 (38.8%)	267
rehabilitation and therapy for the caregiver, e.g., physiotherapist, orthopaedist, etc.	15 (17.6)	14
psychiatric drug therapy	15 (17.6)	19
**Total costs reimbursed by the National Health Fund and the state budget**	85 (100%)	46,928
**Average annual out-of-pocket healthcare expenditure in 2024 (January 1**,** 2024–December 31**,** 2024)**		
***Out-of-pocket direct medical costs for an AS patient***		
non-reimbursed medications	63 (74.1%)	713
supplements	83 (97.6%)	982
specialists’ visits, e.g., dentist, geneticist, neurologist, psychiatrist	69 (81.2%)	903
rehabilitation and therapy	75 (88.2%)	7,836
rehabilitation camps	47 (55.3%)	4,451
other expenses, e.g., special food, special clothing, bibs, legal fees for care, medical toys, etc.	71 (83.5%)	2,154
paid professional care, e.g., nurse, social worker, disability assistant, etc.)	16 (18.8%)	482
medical equipment, e.g., wheelchairs, medical beds, walking frames, walkers, standers, orthoses, orthopaedic shoes, etc.	73 (85.9%)	2,637
car customization/purchase	32 (37.6%)	1,269
**Out-of-pocket direct medical costs for the caregiver**		
psychiatrist or psychologist support for the caregiver	28 (32.9)	617
rehabilitation and therapy for the caregiver, e.g., physiotherapist, orthopaedist, etc.	42 (49.4)	536
psychiatric drug therapy	13 (15.3)	212
**Out-of-pocket indirect costs**		
transport costs/travel expenses to hospital/examinations, rehabilitation	75 (88.2%)	2,126
costs of parents’ stay during hospitalization	29(34.1%)	224
extra electricity, heating, water	76 (89.4%)	447
lost income due to job resignation	56 (65.9%)	31,809
value of informal care (family, neighbours)	41 (48.2%)	6,207
home modifications	46 (54.1%)	4,292
non-reimbursed caregiver support	50 (58.8%)	1,109
additional insurance	34 (40%)	505
**Total private expenditures**	85 (100%)	69,511

Table 5 presents Spearman’s rho correlations between the age of individuals with AS and the utilization or cost of various health services. Younger age was significantly associated with more frequent hospitalizations (ρ = − 0.430, *p* < .001), while older individuals were more likely to receive reimbursed professional care from the NFZ (ρ = 0.268, *p* < .05).

Among out-of-pocket expenses, older age correlated with lower spending on private doctor visits (ρ = − 0.269, *p* < .05), private rehabilitation (ρ = − 0.370, *p* < .001), transportation (ρ = − 0.296, *p* < .01), parental accommodation (ρ = − 0.246, *p* < .05), and informal care (ρ = − 0.271, *p* < .05), reflecting a shift in caregiving patterns and care intensity over time. In contrast, home modification costs increased with age (ρ = 0.252, *p* < .05), suggesting these adaptations accumulate or become more necessary as children grow older.

No significant age-related correlations were found for other categories, such as supplements, medical equipment, or therapeutic camps. Overall, age emerged as a key factor influencing care patterns and related costs in AS.

**Table 5:** Spearman’s Rho correlation between patients’ age and the utilization or cost of healthcare services (reimbursed and out-of-pocket)

		ρ	95%CI	*p*
Patient’s age	hospital stays	-0.430	-0.593; -0.232	**< 0.001**
Patient’s age	reimbursed medications	0.013	-0.225; 0.249	ns
Patient’s age	reimbursed specialists	-0.165	-0.404; 0.095	ns
Patient’s age	reimbursed rehabilitation	0.130	-0.124; 0.368	ns
Patient’s age	reimbursed care	0.268	0.056; 0.457	**< 0.05**
Patient’s age	reimbursed therapeutic camps	0.038	-0.182; 0.254	ns
Patient’s age	reimbursed equipment costs	0.154	-0.079; 0.371	ns
Patient’s age	non-reimbursed medications	-0.061	-0.272; 0.155	ns
Patient’s age	supplements	-0.083	-0.292; 0.134	ns
Patient’s age	private doctor visits	-0.269	-0.457; -0.0.58	**< 0.05**
Patient’s age	private rehabilitation	-0.370	-0.541; -0.169	**< 0.001**
Patient’s age	private therapeutic camps	-0.137	-0.334; 0.091	ns
Patient’s age	other expenses	-0.152	-0.357; 0.067	ns
Patient’s age	paid care	-0.153	-0.356; 0.063	ns
Patient’s age	private medical equipment	-0.127	-0.334; 0.091	ns
Patient’s age	transportation costs	-0.296	-0.481; -0.086	**< 0.01**
Patient’s age	parent accommodation expenses	-0.246	-0.439; -0.032	**< 0.05**
Patient’s age	value of informal care	-0.271	-0.459; -0.061	**< 0.05**
Patient’s age	home modifications	0.252	0.037; 0.444	**< 0.05**

Statistically significant differences are written in boldface; ns: not significant

## Discussion

The results of this study highlight the substantial financial burden faced by families of individuals with AS, underscoring systemic gaps and the urgent need for targeted policy interventions. While previous studies have identified financial well-being as the primary factor linked to reduced quality of life and increased burden among AS caregivers [33, 41], our findings demonstrate that the caregiving demands themselves constitute a significant source of financial strain for families. Out-of-pocket expenses significantly exceed public healthcare reimbursements, a disparity further exacerbated by frequent income loss due to job resignation. Although a significant proportion of parents receive financial support from the government and local government institutions, these funds do not compensate for the lost income caused by the inability to work. This underscores the pronounced financial vulnerability and systemic under-support experienced by affected families. A major cost of caring for an individual with AS is the school care provided by the state in special needs institutions. Additionally, the findings indicate that a patient’s age significantly affects healthcare utilization and related costs. Younger individuals incur more hospitalizations and private rehabilitation expenses, whereas older individuals receive more reimbursed care and generate higher out-of-pocket costs for home adaptations. This suggests that care needs and their economic consequences evolve over time, and that different life stages may have varying impacts on the financial situation of caregivers. Similarly, reimbursed expenditures also shift with the patient’s age, resulting in a temporally uneven distribution of costs covered by public funds.

These findings align with global data on rare diseases’ economic burden. In 2019, 379 rare diseases in the U.S. cost $997 billion, with direct medical costs at $449 billion (45%). Hospitalizations (32%) and prescription drugs (18%) were major direct costs. Indirect costs from productivity loss totaled $437 billion (44%), including absenteeism and caregiving ($149 billion, 34%), presenteeism ($138 billion, 32%), and early retirement ($136 billion, 31%). Non-medical costs were $73 billion (7%), uninsured expenses $38 billion (4%). Average annual medical cost per patient was $28,913, highest in children ($32,037), lowest in those over 65 ($27,157) [46]. Another U.S. study of 24 rare diseases (584,000 patients) estimated $125 billion annual societal costs; average per patient $266,000 (10 times common diseases’ $26,000). Highest costs: metabolic ($334,000) and neurological ($317,000). Lack of treatment increased costs by 21.2% (2.2%–51.8%). Direct costs: $63,000 with treatment vs. $118,000 without. Non-medical costs (5–10%), indirect costs $40,000 with treatment and $73,000 without, making up 40–45% of the total. Mortality costs: $36,000 with treatment, $49,000 without. Total cost for 8.4 million U.S. rare disease patients estimated at $2.2 trillion annually vs. $3.4 trillion for 133 million with common diseases [48]. However, expanding the scope beyond Western countries, similar patterns of economic burden are reported in other regions. For example, a study of 16,945 individuals (8,454 adults, 8,491 children) with 33 rare diseases in China reported annual costs as a percentage of household income: direct medical 105.12%/103.22%, direct non-medical 40.82%/45.30%, indirect 20.98%/22.86% (adults/children). Most experienced high burden (70.2% adults, 57.3% children), followed by low burden (28.7% adults, 42.5% children) and extremely high burden (1.1% adults, 0.2% children). Risk factors for very high burden included older age, poverty, low socioeconomic status, delayed diagnosis, comorbidities, and active treatment; poverty increased the odds of very high burden up to OR 50.37 in adults [49].

Our findings confirm that AS imposes significant economic strain, stemming from both direct and indirect costs of lifelong caregiving. The highest reimbursed medical expenses were hospitalizations ($9,162 per patient annually) and rehabilitation or therapy ($3,159), while the most burdensome out-of-pocket costs were therapy and rehabilitation ($7,836) and medical equipment ($2,637). This is in line with a recent study by Jarvis et al., conducted among 105 caregivers of individuals with AS in the United States, which reported a mean annual economic burden of $79,837 per household (SD = $55,505). Healthcare expenses averaged $827 (SD = $2,072), including $399 for pharmaceuticals (SD = $1,123) and $428 for services (SD = $1,169). Out-of-pocket costs constituted a significant portion of the overall burden, averaging $29,680 (SD = $47,753), with major expenses for vehicle purchases or modifications ($6,717; SD = $17,791), professional care ($6,123; SD = $17,335), home modifications or repairs ($4,387; SD = $15,734), and supportive therapies, i.e. physical or speech therapy ($3,269; SD = $7,564). Additionally, lost leisure time was valued at $6,634 (SD = $4,652) [41].

However, similar financial strains have also been observed among parents of children with other rare neurological disorders. For instance, in EU5 countries, the annual direct medical cost per patient with Dravet syndrome was estimated at nearly $16,000, with approximately 50% attributed to seizure-related symptoms [45]. In Germany, direct costs were estimated at €6,506, with 76% related to hospitalizations, transport, and outpatient care, and only 24% attributed to medications. However, the introduction of newer treatments like stiripentol raised medication costs to 55.4% of total direct costs [44]. Similarly, Elliott et al. found that drug costs represented 72% of overall direct treatment expenses [50]. U.S. estimates showed direct costs exceeding $27,000, largely due to hospitalizations (42%), while indirect costs reached up to $80,000 annually [51]. Another German study reported total direct healthcare costs of approximately €20,000 and indirect costs around €18,000 [52]. Irwin et al. calculated annual costs at €24,944 in direct expenditures, along with €17,594 in maternal and €1,565 in paternal indirect costs [53]. National studies also illustrate significant variability in costs related to specific rare diseases. A Polish study on adult cystic fibrosis patients estimated the average annual treatment cost at €900,729.78 (€19,581.08 per patient), with 70% direct and 30% indirect costs. Of the direct costs, 74% (€467,876.66; €10,171.23 per patient) were for pharmacotherapy, 21% (€132,402.94; €2,878.33 per patient) for hospitalization, with diagnostics (€28,677.93; €623.43 per patient) and outpatient visits (€3,098.03; €65.76 per patient) comprising the remainder. Indirect costs due to productivity loss totaled €262,478.43 (€5,706.03 per patient), and absenteeism costs were €3,536.00 (€76.89 per patient). Notably, indirect costs in Poland exceeded those in most other countries [54].

Finally, these economic burdens translate into significant societal and personal consequences, particularly impacting caregiver employment and productivity. Since more than half of the parents enrolled in this study reported unemployment resulting from caregiving, this study aligns with previous studies showing that high medical costs in AS are further compounded by frequent income loss due to job resignation among caregivers. An Australian study estimated that over 10 years, parents of individuals with AS lost 38.4% of their productivity-adjusted life years (PALYs), totaling 495.06 PALYs and a societal cost of AUD $45.3 million (USD $30 million). Significantly, mothers bore 63% of the burden due to reduced employment and workforce participation, losing 311.66 PALYs (1.50/person; 53.1%) vs. 183.40 PALYs for fathers (1.18/person; 25.2%). This translated into average productivity losses of AUD $145,007 per mother and AUD $128,252 per father. These losses were compounded by caregivers’ heightened physical, mental, and emotional strain [39]. Similarly, a recent study by Jarvis et al. found the average annual cost of AS caregiving to be nearly $80,000, with 53% (~$43,000) due to productivity loss. Job-related impacts were reported by 83.8% of caregivers: 42.9% reduced hours, 35.2% left work for over a year, 24.8% couldn’t take a job, and 21.9% worked irregular hours [41].

Notably, in other rare neurogenetic disorders, elevated indirect costs, driven by job loss, reduced working hours, missed workdays, and loss of income, have also been identified as key predictors of parental depression and reduced quality of life. In France, among 85 parents of children with DS who reported employment difficulties, 33.3% were unemployed, and few worked full-time. Over 50% of mothers had interrupted their employment for more than six months, compared with just 7.1% of fathers [55]. Similarly, Campbell et al. found that 45% of caregivers had quit, retired early, or lost their jobs due to caregiving demands, and 18% had to change jobs [56]. Lagae et al. reported that 80% of EU5 caregivers experienced negative career impacts; among those unemployed (34%), 81% cited caregiving as the main reason. Among employed caregivers, 65% had missed work in the past month, with 28% missing more than three days [45]. Finally, 72% of Polish parents of children with DS reported work restrictions resulting from caregiving [57].

Importantly, mothers reported significantly greater productivity losses than fathers, reflecting a pronounced gender disparity in the caregiving burden. In France, over 50% of mothers had taken employment breaks longer than six months, compared with 7.1% of fathers [55]. In Germany, 31% of mothers quit their jobs (vs. 1% of fathers), 29% reduced working hours (vs. 6% of fathers), and 40% missed work in the preceding three months due to caregiving demands (vs. 27% of fathers) [52].

### Limitations

While this study is the first effort in Poland to assess the self-reported economic burden of caring for individuals with AS, it is not without limitations. Firstly, the sample size of 85 respondents is relatively small. However, given the rarity of AS and the absence of a national rare disease registry, the exact number of affected families in Poland remains unknown. Moreover, considering that only 145 Polish individuals with AS are currently registered in the Global Angelman Syndrome Registry, the 85 household responses collected can be regarded as substantial. Another limitation stems from the retrospective nature of the study and the reliance on subjective caregiver reports, which could not be independently verified. Nevertheless, information about patients’ utilization of healthcare services and associated NFZ-reimbursed expenses was retrieved via the official Internet Patient Account (IKP). At the same time, it should be acknowledged that the IKP system, like any database, may contain occasional inaccuracies. Additionally, estimates of privately financed expenses may be prone to recall errors or incomplete records kept by some respondents. Additionally, household estimates of increased utility use (electricity, heating, water) were based solely on caregiver self-report, which may involve subjective judgment and recall limitations. Objective metering data were not available, and this constraint may introduce some imprecision into the calculation of these indirect costs. The web-based format of the survey could have also influenced participation; however, due to the absence of a centralized registry, recruitment through the patient organization FAST Poland, primarily using its website, social media, and mailing list, was the most feasible approach. Furthermore, the survey did not collect data on absenteeism (i.e., workdays or hours missed due to caregiving), presenteeism, or reduced workforce participation short of full resignation. These important dimensions of productivity loss fell outside the scope of the present study, partly due to the regulatory context in Poland until 2023, in which caregiver benefits were available only to individuals who fully resigned from employment. As a result, our ability to capture the full spectrum of labour-force impacts is limited. In addition, the survey did not collect data on the frequency of specialist care appointments, but only whether such consultations occurred within the previous year, which limits the ability to assess the intensity of specialist care use. An additional limitation of the study is the notable predominance of female respondents, which may introduce gender bias and limit the generalizability of the findings. However, it should be noted that the data were collected at the household level, meaning each response represents the entire family unit rather than just the individual respondent. Additionally, women are more often the primary caregivers and are significantly more likely to manage the household budget. Finally, no methodologically comparable estimates of the financial cost of raising a neurotypical child are available in Poland, which prevents direct benchmarking of the observed economic burden and further highlights the absence of national frameworks for evaluating family-level expenditures.

## Conclusions

This study demonstrates a clear need for continuous, multidisciplinary medical and therapeutic support, with particularly high reliance on neurological, rehabilitative, and communication services. At the same time, families affected by AS face substantial caregiving demands, predominantly carried out by family members, with limited access to formal or informal support and a heavy dependence on unpaid care. The findings highlight the significant productivity burden associated with caregiving, especially for mothers, and emphasize the need for systemic recognition of these often-overlooked economic impacts. Altogether, the results underscore the profound and multifaceted burden AS places on families, not only through direct and indirect financial costs but also through substantial personal and professional consequences. These findings reveal critical gaps in current support systems and call for targeted policy and service-level interventions.

## Data Availability

The data underlying this study’s findings cannot be shared publicly due to the sensitivity and privacy of the study participants. The data will be shared at a reasonable request by the corresponding author.

## Abbreviations

AS: Angelman Syndrome
FAST Poland: Fundacja Foundation for Angelman Syndrome Therapeutics
IKP: Internetowe Konto Pacjenta (Internet Patient Account)
NFZ: Narodowy Fundusz Zdrowia (National Health Fund)

## References

[1] Duis J, Nespeca M, Summers J, Bird L, Bindels-de Heus KGCB, Valstar MJ, et al. A multidisciplinary approach and consensus statement to Establish standards of care for Angelman syndrome. Mol Genet Genomic Med. 2022;10:e1843. 10.1002/mgg3.1843. 35150089 10.1002/mgg3.1843 PMC8922964

[2] Buiting K, Williams C, Horsthemke B. Angelman syndrome — insights into a rare neurogenetic disorder. Nat Rev Neurol Nat Publishing Group. 2016;12:584–93. 10.1038/nrneurol.2016.133. 10.1038/nrneurol.2016.133 27615419

[3] Tones M, Zeps N, Wyborn Y, Smith A, Barrero RA, Heussler H, et al. Does the registry speak your language? A case study of the global Angelman syndrome registry. Orphanet J Rare Dis. 2023;18:330. 10.1186/s13023-023-02904-1. 37858180 10.1186/s13023-023-02904-1 PMC10588126

[4] Arregui Rementeria M, Cadarette S, Oladapo T, Wissinger E, Ruiz K, Kistler K. EPH31 systematic literature review on the global epidemiology of Angelman syndrome. Value Health. 2023;26:S208. 10.1016/j.jval.2023.09.1073.

[5] Strona główna - Globalny rejestr zespołu Angelmana [Internet]. [cited 2025 Mar 27]. https://www.angelmanregistry.info/pl/. Accessed 27 Mar 2025.

[6] Miodrag N, Peters S. Parent stress across molecular subtypes of children with Angelman syndrome. J Intellect Disabil Res. 2015;59:816–26. 10.1111/jir.12195. 25833412 10.1111/jir.12195

[7] Mertz LGB, Thaulov P, Trillingsgaard A, Christensen R, Vogel I, Hertz JM, et al. Neurodevelopmental outcome in Angelman syndrome: genotype-phenotype correlations. Res Dev Disabil. 2014;35:1742–7. 10.1016/j.ridd.2014.02.018. 24656292 10.1016/j.ridd.2014.02.018

[8] Yang L, Shu X, Mao S, Wang Y, Du X, Zou C. Genotype-Phenotype correlations in Angelman syndrome. Genes (Basel). 2021;12:987. 10.3390/genes12070987. 34203304 10.3390/genes12070987 PMC8304328

[9] Thibert RL, Larson AM, Hsieh DT, Raby AR, Thiele EA. Neurologic manifestations of Angelman syndrome. Pediatr Neurol. 2013;48:271–9. 10.1016/j.pediatrneurol.2012.09.015. 23498559 10.1016/j.pediatrneurol.2012.09.015

[10] Suleja A, Milska-Musa K, Przysło Ł, Bednarczyk M, Kostecki M, Cysewski D, et al. Angelman syndrome in poland: current diagnosis and therapy status-the caregiver perspective: a questionnaire study. Orphanet J Rare Dis. 2024;19:306. 10.1186/s13023-024-03292-w. 39174987 10.1186/s13023-024-03292-w PMC11340045

[11] Williams CA. The behavioral phenotype of the Angelman syndrome. Am J Med Genet C Semin Med Genet. 2010;154 C:432–7. 10.1002/ajmg.c.30278. 20981772 10.1002/ajmg.c.30278

[12] Samanta D. Epilepsy in Angelman syndrome: a scoping review. Brain Dev. 2021;43:32–44. 10.1016/j.braindev.2020.08.014. 32893075 10.1016/j.braindev.2020.08.014 PMC7688500

[13] Clayton-Smith J, Laan L. Angelman syndrome: a review of the clinical and genetic aspects. J Med Genet. 2003;40:87–95. 10.1136/jmg.40.2.87. 12566516 10.1136/jmg.40.2.87 PMC1735357

[14] Williams CA, Driscoll DJ, Dagli AI. Clinical and genetic aspects of Angelman syndrome. Genet Med. 2010;12:385–95. 10.1097/GIM.0b013e3181def138. 20445456 10.1097/GIM.0b013e3181def138

[15] Williams CA, Beaudet AL, Clayton-Smith J, Knoll JH, Kyllerman M, Laan LA, et al. Angelman syndrome 2005: updated consensus for diagnostic criteria. Am J Med Genet A. 2006;140:413–8. 10.1002/ajmg.a.31074. 16470747 10.1002/ajmg.a.31074

[16] Wheeler AC, Sacco P, Cabo R. Unmet clinical needs and burden in Angelman syndrome: a review of the literature. Orphanet J Rare Dis. 2017;12:164. 10.1186/s13023-017-0716-z. 29037196 10.1186/s13023-017-0716-z PMC5644259

[17] Tan W-H, Bird LM, Thibert RL, Williams CA. If not Angelman, what is it? A review of Angelman-like syndromes. Am J Med Genet A. 2014;164A:975–92. 10.1002/ajmg.a.36416. 24779060 10.1002/ajmg.a.36416

[18] Welham A, Lau J, Moss J, Cullen J, Higgs S, Warren G, et al. Are Angelman and Prader-Willi syndromes more similar than we thought? Food-related behavior problems in Angelman, Cornelia de Lange, fragile X, Prader-Willi and 1p36 deletion syndromes. Am J Med Genet A. 2015;167A:572–8. 10.1002/ajmg.a.36923. 25691410 10.1002/ajmg.a.36923

[19] Williams CA, Lossie A, Driscoll D, Phillips RC, Unit. Angelman syndrome: mimicking conditions and phenotypes. Am J Med Genet. 2001;101:59–64. 10.1002/ajmg.1316. 11343340 10.1002/ajmg.1316

[20] Roche L, Tones M, Williams MG, Cross M, Simons C, Heussler H. Caregivers report on the pathway to a formal diagnosis of Angelman syndrome: A comparison across genetic etiologies within the global Angelman syndrome registry. Adv Neurodev Disord. 2021;5:193–203. 10.1007/s41252-021-00195-w.

[21] Dagli A, Buiting K, Williams CA. Molecular and clinical aspects of Angelman syndrome. Mol Syndromol. 2012;2:100–12. 10.1159/000328837. 22670133 10.1159/000328837 PMC3366701

[22] Copping NA, McTighe SM, Fink KD, Silverman JL. Emerging gene and small molecule therapies for the neurodevelopmental disorder Angelman syndrome. Neurotherapeutics. 2021;18:1535–47. 10.1007/s13311-021-01082-x. 34528170 10.1007/s13311-021-01082-x PMC8608975

[23] Heussler HS. Emerging therapies and challenges for individuals with Angelman syndrome. Curr Opin Psychiatry. 2021;34:123–8. 10.1097/YCO.0000000000000674. 33395098 10.1097/YCO.0000000000000674

[24] Keary CJ, McDougle CJ. Current and emerging treatment options for Angelman syndrome. Expert Rev Neurother. 2023;23:835–44. 10.1080/14737175.2023.2245568. 37599585 10.1080/14737175.2023.2245568

[25] Domínguez-Berjón MF, Zoni AC, Esteban-Vasallo MD, Sendra-Gutiérrez JM, Astray-Mochales J. Main causes of hospitalization in people with Angelman syndrome. J Appl Res Intellect Disabil. 2018;31:466–9. 10.1111/jar.12411. 28869323 10.1111/jar.12411

[26] Coppus AMW. People with intellectual disability: what do we know about adulthood and life expectancy? Dev Disabil Res Rev. 2013;18:6–16. 10.1002/ddrr.1123. 23949824 10.1002/ddrr.1123

[27] Herbst J, Byard RW. Sudden death and Angelman syndrome. J Forensic Sci. 2012;57:257–9. 10.1111/j.1556-4029.2011.01901.x. 21854386 10.1111/j.1556-4029.2011.01901.x

[28] Gomes AT, Moore A, Cross M, Hardesty C, David K, Sampedro MG, et al. Community-Sourced reporting of mortalities in Angelman syndrome (1979–2022). Am J Med Genet Part A. 2025;197:e63961. 10.1002/ajmg.a.63961. 39679858 10.1002/ajmg.a.63961 PMC12166961

[29] Griffith GM, Hastings RP, Oliver C, Howlin P, Moss J, Petty J, et al. Psychological well-being in parents of children with Angelman, Cornelia de Lange and cri du chat syndromes. J Intellect Disabil Res. 2011;55:397–410. 10.1111/j.1365-2788.2011.01386.x. 21323782 10.1111/j.1365-2788.2011.01386.x

[30] van den Borne HW, van Hooren RH, van Gestel M, Rienmeijer P, Fryns JP, Curfs LM. Psychosocial problems, coping strategies, and the need for information of parents of children with Prader-Willi syndrome and Angelman syndrome. Patient Educ Couns. 1999;38:205–16. 10.1016/s0738-3991(99)00004-x. 10865686 10.1016/s0738-3991(99)00004-x

[31] Wulffaert J, Scholte EM, Van Berckelaer-Onnes IA. Maternal parenting stress in families with a child with Angelman syndrome or Prader-Willi syndrome. J Intellect Dev Disabil. 2010;35:165–74. 10.3109/13668250.2010.499101. 20809878 10.3109/13668250.2010.499101

[32] Thomson A, Glasson E, Roberts P, Bittles A. Over time it just becomes easier… parents of people with Angelman syndrome and Prader-Willi syndrome speak about their carer role. Disabil Rehabil. 2017;39:763–70. 10.3109/09638288.2016.1161838. 27015406 10.3109/09638288.2016.1161838

[33] Walkowiak D, Domaradzki J. Caregiving burden and quality of life among parents of individuals with Angelman syndrome: gender differences and the impact of financial Well-Being. Pediatr Neurol. 2025;169:31–9. 10.1016/j.pediatrneurol.2025.05.005. 40449417 10.1016/j.pediatrneurol.2025.05.005

[34] Griffith GM, Hastings RP, Nash S, Petalas M, Oliver C, Howlin P, et al. You have to sit and explain it All, and explain Yourself. Mothers’ experiences of support services for their offspring with a rare genetic intellectual disability syndrome. J Genet Couns. 2011;20:165–77. 10.1007/s10897-010-9339-4. 21203808 10.1007/s10897-010-9339-4

[35] Walkowiak D, Domaradzki J. Perception of psychosocial burden in mothers of children with rare pediatric neurological diseases. Sci Rep. 2025;15:6295. 10.1038/s41598-025-87251-w. 39984547 10.1038/s41598-025-87251-w PMC11845487

[36] Adams D, Clarke S, Griffith G, Howlin P, Moss J, Petty J, et al. Mental health and Well-Being in mothers of children with rare genetic syndromes showing chronic challenging behavior: A Cross-Sectional and longitudinal study. Am J Intellect Dev Disabil. 2018;123:241–53. 10.1352/1944-7558-123.3.241. 29671635 10.1352/1944-7558-123.3.241

[37] Adams D, Hastings RP, Alston-Knox C, Cianfaglione R, Eden K, Felce D, et al. Using bayesian methodology to explore the profile of mental health and well-being in 646 mothers of children with 13 rare genetic syndromes in relation to mothers of children with autism. Orphanet J Rare Dis. 2018;13:185. 10.1186/s13023-018-0924-1. 30359268 10.1186/s13023-018-0924-1 PMC6203267

[38] Willgoss T, Cassater D, Connor S, Krishnan ML, Miller MT, Dias-Barbosa C, et al. Measuring what matters to individuals with Angelman syndrome and their families: development of a Patient-Centered disease concept model. Child Psychiatry Hum Dev. 2021;52:654–68. 10.1007/s10578-020-01051-z. 32880036 10.1007/s10578-020-01051-z PMC8238699

[39] Sansom SL, Baker EK, Godler DE, Liew D. Estimating the impact of Angelman syndrome on parental productivity in Australia using productivity-adjusted life years. Disabil Health J. 2023;16:101423. 10.1016/j.dhjo.2022.101423. 36639256 10.1016/j.dhjo.2022.101423

[40] Mathur S, Premshekar A, Aggarwal P, Gupta J, Siddiqui M. EE591 global health economic burden of Angelman syndrome: a systematic literature review. Value Health. 2024;27:S171. 10.1016/j.jval.2024.10.871.

[41] Jarvis J, Chertavian E, Buessing M, Renteria T, Tu L, Hoffer L, et al. The economic impact of caregiving for individuals with Angelman syndrome in the united states: results from a caregiver survey. Orphanet J Rare Dis. 2025;20:82. 10.1186/s13023-025-03551-4. 39985061 10.1186/s13023-025-03551-4 PMC11846284

[42] Nowak JK, Walkowiak J. Study designs in medical research and their key characteristics. J Med Sci. 2023;92:e928–928. 10.20883/medical.e928.

[43] Frisch N, Atherton P, Doyle-Waters MM, MacLeod MLP, Mallidou A, Sheane V, et al. Patient-oriented research competencies in health (PORCH) for researchers, patients, healthcare providers, and decision-makers: results of a scoping review. Res Involv Engagem. 2020;6:4. 10.1186/s40900-020-0180-0. 32055415 10.1186/s40900-020-0180-0 PMC7011284

[44] Strzelczyk A, Schubert-Bast S, Reese JP, Rosenow F, Stephani U, Boor R. Evaluation of health-care utilization in patients with Dravet syndrome and on adjunctive treatment with stiripentol and clobazam. Epilepsy Behav. 2014;34:86–91. 10.1016/j.yebeh.2014.03.014. 24727467 10.1016/j.yebeh.2014.03.014

[45] Lagae L, Irwin J, Gibson E, Battersby A. Caregiver impact and health service use in high and low severity Dravet syndrome: A multinational cohort study. Seizure. 2019;65:72–9. 10.1016/j.seizure.2018.12.018. 30616222 10.1016/j.seizure.2018.12.018

[46] Yang G, Cintina I, Pariser A, Oehrlein E, Sullivan J, Kennedy A. The National economic burden of rare disease in the united States in 2019. Orphanet J Rare Dis. 2022;17:163. 10.1186/s13023-022-02299-5. 35414039 10.1186/s13023-022-02299-5 PMC9004040

[47] World Economic Outlook. (April 2025) - Implied PPP conversion rate [Internet]. [cited 2025 June 17]. https://www.imf.org/external/datamapper/PPPEX@WEO. Accessed 17 June 2025.

[48] Andreu P, Karam J, Child C, Chiesi G, Cioffi G. The burden of rare diseases: an economic evaluation.

[49] Yu J, Chen S, Zhang H, Zhang S, Dong D. Patterns of the health and economic burden of 33 rare diseases in china: nationwide Web-Based study. JMIR Public Health Surveill. 2024;10:e57353. 10.2196/57353. 39190906 10.2196/57353 PMC11387910

[50] Elliott J, McCoy B, Clifford T, Wells GA, Coyle D. Economic evaluation of stiripentol for Dravet syndrome: A Cost-Utility analysis. PharmacoEconomics. 2018;36:1253–61. 10.1007/s40273-018-0669-7. 29761351 10.1007/s40273-018-0669-7

[51] Whittington MD, Knupp KG, Vanderveen G, Kim C, Gammaitoni A, Campbell JD. The direct and indirect costs of Dravet syndrome. Epilepsy Behav. 2018;80:109–13. 10.1016/j.yebeh.2017.12.034. 29414539 10.1016/j.yebeh.2017.12.034

[52] Strzelczyk A, Kalski M, Bast T, Wiemer-Kruel A, Bettendorf U, Kay L, et al. Burden-of-illness and cost-driving factors in Dravet syndrome patients and carers: A prospective, multicenter study from Germany. Eur J Paediatr Neurol. 2019;23:392–403. 10.1016/j.ejpn.2019.02.014. 30871879 10.1016/j.ejpn.2019.02.014

[53] Irwin J, Strzelczyk A, Kalski M, Bast T, Wiemer-Kruel A, Bettendorf U, et al. PND78 - Socio-economic impact of Dravet syndrome in Germany: a real-world study. Value in health. Elsevier. 2018;21:S342. 10.1016/j.jval.2018.09.2044.

[54] Kopciuch D, Zaprutko T, Paczkowska A, Nowakowska E. Costs of treatment of adult patients with cystic fibrosis in Poland and internationally. Public Health. 2017;148:49–55. 10.1016/j.puhe.2017.03.003. 28404533 10.1016/j.puhe.2017.03.003

[55] Nabbout R, Dirani M, Teng T, Bianic F, Martin M, Holland R, et al. Impact of childhood Dravet syndrome on care givers of patients with DS, a major impact on mothers. Epilepsy Behav. 2020;108:107094. 10.1016/j.yebeh.2020.107094. 32375095 10.1016/j.yebeh.2020.107094

[56] Campbell JD, Whittington MD, Kim CH, VanderVeen GR, Knupp KG, Gammaitoni A. Assessing the impact of caring for a child with Dravet syndrome: results of a caregiver survey. Epilepsy Behav. 2018;80:152–6. 10.1016/j.yebeh.2018.01.003. 29414545 10.1016/j.yebeh.2018.01.003

[57] Domaradzki J, Walkowiak D. Caring for children with Dravet syndrome: exploring the daily challenges of family caregivers. Child (Basel). 2023;10:1410. 10.3390/children10081410. 10.3390/children10081410 PMC10453293 37628409

